# Supplementary material for: Strengthening diagnostic capacity for viral hemorrhagic fevers in Forest Guinea: advances in Lassa fever case detection
Source: Npj Viruses. 2026 Sep 16;4:42. doi: 10.1038/s44298-026-00239-9 (PMC13582950; doi:10.1038/s44298-026-00239-9)
Supplement: Supplementary file 1 [file 44298_2026_239_MOESM1_ESM.docx]

**-Supplementary file**

**Title:**

Strengthening diagnostic capacity for viral hemorrhagic fevers in Forest Guinea: advances in Lassa fever case detection

**Authors**

Fara Raymond Koundouno ^1,*^, Youssouf Sidibe ^2,*^, Saa Lucien Millimono ^1^, Kékoura Ifono ^1^, Julia Hinzmann ^3^, Hugo Soubrier ^3^, Karifa Kourouma ^1^, Tamba Elie Millimouno ^1^, Fernand M’Bemba Tolno ^1^, Faya Moriba Kamano ^1^, Mamadou Dioulde Barry ^2^, Soua Koulemou ^2^, Bely Sonomy ^2^, Mariame Traore ^2^, Kaba Keïta ^2^, Mette Hinrichs ^3^, Sarah Ryter ^3^, Carolina van Gelder ^3^, Beate Becker-Ziaja ^3^, Christine Jacobsen ^3^, Anke Thielebein ^3^, Lisa Oestereich ^3^, Meike Pahlmann ^3^, Anaïs Legand ^4^, Pierre Formenty ^4^, Aly Antoine Kamano ^5^, Seydou Dia ^5^, Moke Fundji Jean Marie Kipela 5^6^, Stephan Günther ^3^, Moussa Kolié ^6^, Kaba Keïta ^7^, Sira Hélène Guilavogui ^8^, Sanaba Boumbaly ^9^, N’Faly Magassouba ^9,†^, Sophie Duraffour ^3, †^, Giuditta Annibaldis ^3, †,🖂^

* contributed equally

^†^ contributed equally

^🖂^ corresponding author: Giuditta Annibaldis, Bernhard Nocht Institute for Tropical Medicine (BNITM), Bernhard Nocht Straße 74, 20359, Hamburg, Germany; giuditta.annibaldis@bnitm.de, phone: +49 40 285380 932

Table of Contents

[Table S1	2](#_Toc237018634)

[Table S2	3](#_Toc237018635)

[Table S3	4](#_Toc237018636)

[Table S4	5](#_Toc237018637)

[Table S5	6](#_Toc237018638)

[Table S6	7](#_Toc237018639)

[Table S7	8](#_Toc237018640)

[Table S8	9](#_Toc237018641)

[Table S9	10](#_Toc237018642)

[Figure S1	11](#_Toc237018643)

## Table S1

**VHFs diagnostics testing performed at LFHV-GKD, 2017-2024**

| **Pathogen /Year** | **n positive / N tested (% positive) *** | | | | | | | | | |
| --- | --- | --- | --- | --- | --- | --- | --- | --- | --- | --- |
|  | **2017** | **2018** | **2019** | | **2020** | **2021** | **2022** | **2023** | **2024** | **All years** |
| **EBOV** | 0/203  (0.0%) | 0/6  (0.0%) | 0/157  (0.0%) | | 0/7  (0.0%) | 8/202 (4.0%) | 0/340  (0.0%) | 0/284  (0.0%) | 0/191  (0.0%) | **8/1390 (0.6%)** |
| **MARV** | 0/113  (0.0%) | 0/6  (0.0%) | 0/143  (0.0%) | | 0/7  (0.0%) | 1/202 (0.5%) | 0/340  (0.0%) | 0/284  (0.0%) | 0/191  (0.0%) | **1/1286**  **(0.1%)** |
| **LASV** | 0/106  (0.0%) | 0/39  (0.0%) | 1/171**  (0.6%) | | 2/82  (2.4%) | 1/75 (1.3%) | 3/341  (0.9%) | 8/287  (2.8%) | 3/189  (1.6%) | **18/1290**  **(1.4%)** |
| **YFV** | 0/252  (0.0%) | 0/89  (0.0%) | 0/55  (0.0%) | | 0/39  (0.0%) | 0/2 (0.0%) | 0/64  (0.0%) | 1/72  (1.4%) | 0/53  (0.0%) | **1/626**  **(0.2%)** |
| **DENV** | na | na | 0/14  (0.0%) | | 0/3  (0.0%) | na | 0/69  (0.0%) | 0/62  (0.0%) | 0/53  (0.0%) | **0/201**  **(0.0%)** |
| **Total test performed** | **674** | **140** | **540** | | **138** | **481** | **1154** | **989** | **677** | **4793** |
| **Specimen type** | | | | | | | | | | |
| **Blood** | 513 | 132 | 362 | 152 | | 157 | 336 | 280 | 179 | **2111** |
| **Oral swab** | 3 | 1 | 2 | 7 | | 52 | 5 | 7 | 12 | **89** |
| **Total samples received** | **516** | **133** | **364** | **159** | | **209** | **341** | **287** | **191** | **2200** |

Abbreviations: na, not applicable; EBOV, Ebola virus; MARV, Marburg virus; LASV, Lassa virus; YFV, yellow fever virus; DENV, dengue virus.

The values were used to create Figure 1A-B and 3A (secondary confirmatory tests were excluded).

*Follow up tests performed for patient monitoring were excluded from the count (only initial diagnosis displayed).

**The LF case in 2019 was initially detected by the national reference lab for VHFs in Conakry (CRV-LFHVG), then later confirmed by LFHV-GKD (*Magassouba et al., 2020*).

## Table S2

**Sociodemographic characteristics of VHFs suspected cases tested at LFHV-GKD, 2017-2024**

| **Characteristics** | **Frequency (percentage)** |
| --- | --- |
| **Age (years)****  Median (IQR), min-max: 35 (24-50), 1-95 |  |
| **Sex**  Female  Male | 1252/2200 (56.5%)  958/2200 (43.5%) |
| **Occupation**  Domestic worker  Farmer  Health worker  Other  Student*  Unknown | 762/2200 (34.7%)  337/2200 (15.3%)  75/2200 (3.4%)  603/2200 (27.4%)  357/2200 (16.2%)  66/2200 (3.0%) |
| **Prefecture of residence****  Guéckédou  N’Zérékoré  Other  Yomou | 2099/2192 (95.8%)  31/2192 (1.4%)  34/2192 (1.5%)  28/2192 (1.3%) |

Abbreviations: IQR, Interquartile range.

*Including children in pre-school age (e.g. 3 years old).

**Total number differs from total, missing data.

## Table S3

**VHFs diagnostics testing performed at HRNZE, 2021-2024**

| **Pathogen /Year** | **n positive / N tested (% positive) *** | | | | |
| --- | --- | --- | --- | --- | --- |
|  | **2021** | **2022** | **2023** | **2024** | **All years** |
| **EBOV** | 14/1972** (0.7%) | 0/70  (0.0%) | 0/172  (0.0%) | 0/133  (0.0%) | **14/2347 (0.6%)** |
| **MARV** | 0/3  (0.0%) | 0/70  (0.0%) | 0/172  (0.0%) | 0/133  (0.0%) | **0/378**  **(0.0%)** |
| **LASV***** | 9/231  (3.9%) | 2/71 (2.8%) | 1/174 (0.6%) | 2/135 (1.5%) | **14/611 (2.3%)** |
| **YFV** | na | 0/12  (0.0%) | 0/91  (0.0%) | 0/83  (0.0%) | **0/186**  **(0.0%)** |
| **DENV** | na | 0/28  (0.0%) | 0/53  (0.0%) | 1/83  (1.2%) | **1/164**  **(0.6%)** |
| **Total test performed** | 2206 | 251 | 662 | 567 | 3686 |
| **Specimen type** | | | | | |
| **Blood** | 1069 | 73 | 174 | 130 | **1466** |
| **Oral swab** | 1030 | 2 | 0 | 5 | **1037** |
| **Total samples received** | **2099** | **75** | **174** | **135** | **2483** |

Abbreviations: na, not applicable; EBOV, Ebola virus; MARV, Marburg virus; LASV, Lassa virus; YFV, yellow fever virus; DENV, dengue virus.

The values in the table were used to create Figure 1C-D and 3A (secondary confirmatory tests were excluded).

*Follow up tests performed for patient monitoring were excluded from the count (only initial diagnosis displayed).

**Includes testing by real time RT-PCR and RDTs; six of the EBOV positive cases had prior positive results at LFHV-GKD (confirmatory testing).

***In 2021, of the 9 LF cases, 7 were detected through prospective testing, while 2 were from retrospective investigation; in 2022 one LF case tested at HRNZE corresponds to a LF case previously detected at LFHV-GKD who sought health care later at the Regional Hospital in N’Zérékoré.

## Table S4

**Sociodemographic characteristics of VHFs suspected cases tested at HRNZE, 2021-2024**

| **Characteristics** | **Frequency (percentage)** |
| --- | --- |
| **Age (years)**  Median (IQR), min-max: 46 (27-67), 1-107 |  |
| **Sex**  Female  Male | 1164/2483 (46.9%)  1319/2483 (53.1%) |
| **Occupation**  Domestic worker  Farmer  Health worker  Other  Student*  Unknown | 625/2483 (25.2%)  264/2483 (10.6%)  102/2483 (4.1%)  704/2483 (28.3%)  742/2483 (29.9%)  46/2483 (1.9%) |
| **Prefecture of residence**  Beyla  Lola  Macenta  N’Zérékoré  Other  Yomou | 108/2483 (4.4%)  122/2483 (4.9%)  87/2483 (3.5%)  2081/2483 (83.8%)  23/2483 (0.9%)  62/2483 (2.5%) |

Abbreviations: IQR, Interquartile range.

*Including children in pre-school age (e.g. 3 years old).

## Table S5

**Monthly number of tests performed and positive pathogen detections at LFHV-GKD and at LFHV-HRNZE**

| **Site** | **LFHV-GKD, 2017-2024** | | | | | **LFHV-HRNZE, 2021-2024** | | | | |
| --- | --- | --- | --- | --- | --- | --- | --- | --- | --- | --- |
| **Pathogen /Month** | **n positive / N tested** | | | | | **n positive / N tested** | | | | |
|  | **EBOV** | **MARV** | **LASV** | **YFV** | **DENV** | **EBOV** | **MARV** | **LASV** | **YFV** | **DENV** |
| **Jan** | 0/89 | 0/85 | 2/118 | 0/110 | 0/16 | 0/28 | 0/28 | 0/28 | 0/2 | 0/5 |
| **Feb** | 8/133 | 0/116 | 2/89 | 0/12 | 0/5 | 11/46 | 0/20 | 1/20 | 0/10 | 0/4 |
| **Mar** | 0/134 | 0/79 | 1/99 | 0/20 | 0/19 | 1/173 | 0/33 | 2/34 | 0/12 | 0/0 |
| **Apr** | 0/139 | 0/125 | 2/131 | 0/21 | 0/13 | 2/268 | 0/26 | 0/26 | 0/11 | 0/18 |
| **May** | 0/134 | 0/134 | 1/117 | 0/40 | 0/25 | 0/399 | 0/33 | 2/52 | 0/32 | 0/34 |
| **Jun** | 0/118 | 0/118 | 1/91 | 0/38 | 0/14 | 0/567 | 0/36 | 3/92 | 0/29 | 0/36 |
| **Jul** | 0/112 | 0/112 | 2/131 | 0/70 | 0/32 | 0/343 | 0/20 | 2/104 | 0/20 | 1/9 |
| **Aug** | 0/156 | 1/156 | 1/136 | 0/31 | 0/7 | 0/226 | 0/30 | 2/56 | 0/28 | 0/9 |
| **Sep** | 0/89 | 0/89 | 5/83 | 0/91 | 0/3 | 0/167 | 0/47 | 1/86 | 0/11 | 0/10 |
| **Oct** | 0/59 | 0/59 | 0/78 | 0/51 | 0/11 | 0/41 | 0/21 | 0/26 | 0/10 | 0/11 |
| **Nov** | 0/121 | 0/107 | 0/112 | 0/65 | 0/27 | 0/38 | 0/33 | 0/34 | 0/13 | 0/9 |
| **Dec** | 0/106 | 0/106 | 1/105 | 1/77 | 0/29 | 0/51 | 0/51 | 1/53 | 0/8 | 0/19 |
| **Total detected/ tested** | **8/**  **1390** | **1/**  **1286** | **18/**  **1290** | **1/**  **626** | **0/**  **201** | **14/**  **2347** | **0/**  **378** | **14/**  **611** | **0/**  **186** | **1/**  **164** |

Abbreviations: EBOV, Ebola virus; MARV, Marburg virus; LASV, Lassa virus; YFV, yellow fever virus; DENV, dengue virus.

## Table S6

**Sociodemographic characteristics of Lassa fever cases, Guinea 2020-2024**

| **Characteristics** | **Frequency (percentage)** |
| --- | --- |
| **Age (years)**  Median (IQR), min-max = 31 (24-50), 3-75  <25 years  25-30 years  31-49 years  >49 years | 8/30 (26.7%)  7/30 (23.3%)  7/30 (23.33%)  8/30 (26.7%) |
| **Sex**  Female  Male | 10/30 (33.3%)  20/30 (66.7%) |
| **Occupation**  Domestic worker  Farmer  Health worker  Other  Student* | 7/30 (23.3%)  7/30 (23.3%)  2/30 (6.7%)  8/30 (26.7%)  6/30 (20.0%) |
| **Prefecture of residence**  Beyla  Faranah  Guéckédou  Kissidougou  Lola  N’Zérékoré  Yomou | 1/30 (3.3%)  1/30 (3.3%)  15/30 (50.0%)  1/30 (3.3%)  2/30 (6.7%)  8/30 (26.7%)  2/30 (6.7%) |
| **Year**  2020  2021**  2022  2023  2024 | 2/30 (6.7%)  10/30 (33.3%)  4/30 (13.3%)  9/30 (30.0%)  5/30 (16.7%) |

Abbreviations: IQR, Interquartile range.

The values were used to create Figure 2.

*Including children in pre-school age (e.g. 3 years old)

**Including two samples from retrospective testing, not notified to MoH

## Table S7

**Symptoms at presentation among Lassa fever cases, Guinea 2020-2024**

| **Symptom*** | **Frequency (percentage)** |
| --- | --- |
| Fever | 27/30 (90%) |
| Fatigue | 22/30 (73%) |
| Vomit | 19/30 (63%) |
| Bleeding | 18/30 (60%) |
| Headache | 18/30 (60%) |
| Abdominal pain | 14/30 (47%) |
| Anorexia | 13/30 (43%) |
| Cough | 8/30 (27%) |
| Diarrhea | 7/30 (23%) |
| Resp. Diff. | 6/30 (20%) |
| Hiccup | 6/30 (20%) |
| Chest pain | 5/30 (17%) |
| Joint pain | 5/30 (17%) |
| Sore throat | 5/30 (17%) |

*As recorded on laboratory request form accompanying the sample.

The values were used to create Figure 3B.

## Table S8

**Outcome, laboratory findings and ribavirin treatment among Lassa fever cases, Guinea 2020-2024**

| **Variable** | **Frequency (percentage)** |
| --- | --- |
| **Duration between symptom onset to sample collection (days)**  Median (IQR), min-max = 7 (3-10), 0-22  <3  3-7  8-10  >10 | 7/29 (24.1%)  10/29 (34.5%)  5/29 (17.3%)  7/29 (24.1%) |
| **Duration between symptom onset to test result (days)**  Median (IQR), min-max = 7 (3-10.5), 0-23  <3  3-7  8-10  >10 | 3/28 (10.7%)  12/28 (42.9%)  6/28 (21.4%)  7/28 (25.0%) |
| **Ct value S-segment**  Median (IQR), min-max = 30 (23-33), 14-44  <19  19-23  24-28  29-33  34-38  39-44  ≥45* | 3/30 (10.0%)  5/30 (16.7%)  5/30 (16.7%)  10/30 (33.3%)  4/30 (13.3%)  2/30 (6.7%)  1/30 (3.3%) |
| **Ct value L-segment**  Median (IQR), min-max = 26.5 (22-31), 16-44  <19  19-23  24-28  29-33  34-38  39-44  ≥45* | 2/30 (6.7%)  8/30 (26.7%)  10/30 (33.3%)  7/30 (23.3%)  2/30 (6.7%)  0/30 (0.0%)  1/30 (3.3%) |
| **Serology status (anti-LASV IgG/IgM)*****  IgM positive  IgG positive | 7/22 (31.8%)  16/22 (72.7%) |
| **Outcome****  Alive  Dead | 9/28 (32.1%)  19/28 (67.9%) |
| **Ribavirin treatment****  Yes  No | 10/28 (35.7%)  18/28 (64.3%) |

Abbreviations: IQR, Interquartile range; Ct, cycle threshold, S-segment, small segment of Lassa virus; L-segment, large segment of Lassa virus; IgM, immunoglobulin M; IgG, immunoglobulin G.

The values were used to create Figure 3C-D

* Negative, as over the limit of detection of real time RT-PCR assay.

** Total number differs due to incomplete data records.

*** Total number differs due to sample volume limitation and or kit access.

## Table S9

**Comparison of demographic and laboratory factors by mortality among Lassa fever cases, Guinea 2020-2024**

| **Variable** | **Frequency (percentage)** | |
| --- | --- | --- |
|  | **Dead** | **Alive** |
| **Sex**  Female  Male | 8/19 (42.1%)  11/19 (57.9%) | 2/9 (22.2%)  7/9 (77.8%) |
| **Age, median years (IQR)** | 35 (25-52) | 25 (22-30) |
| **Prefecture of residence**  Guéckédou  Others | 7/19 (34.8%)  12/19 (63.2%) | 8/9 (88.9%)  1/9 (11.1%) |
| **Duration between symptom onset to sampling, median days (IQR)** | 6 (2-9) | 3 (2-4) |
| **Ct value S-segment, median (IQR)** | 27 (21-31) | 32 (31-34) |
| **Ct value L-segment, median (IQR)** | 25 (20-28) | 28 (26-31) |
| **Serology status (anti-LASV IgM)***  IgM positive  IgM negative | 3/13 (23.1%)  10/13 (76.9%) | 3/7 (42.9%)  4/7 (57.1%) |
| **Serology status (anti-LASV IgG)***  IgG positive  IgG negative | 10/13 (76.9%)  3/13 (23.1%) | 5/7 (71.4%)  2/7 (28.6%) |

Abbreviations: IQR, Interquartile range; Ct, cycle threshold, S-segment, small segment of Lassa virus; L-segment, large segment of Lassa virus; IgM, immunoglobulin M; IgG, immunoglobulin G.

* Total number differs due to serological testing limitation and outcome data missing.

## Figure S1

**Correlation between Ct values for S-segment and L-segment among Lassa fever cases, Guinea 2020-2024**

**Figure S1. Correlation between Ct values for S-segment and L-segment among Lassa fever cases, Guinea 2020-2024.** Scatter plot of real-time RT–PCR Ct values for S-segment (x-axis) and L-segment (y-axis) from LASV–positive samples (n=30). Each point represents a sample (first test only included). A strong positive association was observed (R²=0.7708; p<0.0001). The fitted regression line is shown (Y=0·6999×X+5·900).
